# Epiphysiolysis Type Salter I of the Medial Clavicle with Posterior Displacement: A Case Series and Review of the Literature

**DOI:** 10.1155/2018/4986061

**Published:** 2018-09-27

**Authors:** C. Siebenmann, F. Ramadani, G. Barbier, E. Gautier, P. Vial

**Affiliations:** Department of Orthopedic Surgery, HFR Fribourg-Hôpital Cantonal, Switzerland

## Abstract

Physeal fractures of the medial clavicle with posterior displacement of the metaphysis are very rare injuries, but additional injuries can be life-threatening. Due to the specific clavicular ossification process, skeletally immature patients present usually not true sternoclavicular joint (SCJ) dislocations accordingly to adults but rather displaced physeal fractures. There is no consensus in the current literature on the best treatment of this lesion. Conservative treatment is not resulting in good outcome; closed reduction is often not successful, and open reduction with internal fixation is finally required. Several methods are described for stabilizing these physeal fractures. We treated three osseous immature patients with this lesion. Due to the small dimension of the medial clavicular epiphysis, we performed in one case a transosseous figure-of-eight suture of the clavicular metaphysis towards the sternum, and in the two other cases, a transosseous suture from the clavicular metaphysis on the anterior clavicular periosteum. The latter technique avoids harm to the small epiphysis or the SCJ and minimizes the risk of retrosternal complications.

## 1. Introduction

Lesions of the sternoclavicular joint (SCJ) including epiphysiolyses type Salter I and II are rare in all age groups, representing less than 5% of all shoulder girdle injuries [1]. Posterior dislocations of the medial end of the clavicle in skeletally immature patients are exceptional. But, potential life-threating complications can occur due to the proximity to the trachea, esophagus, and retrosternal vascular and neural structures. In the literature, severe complications are reported resulting from missed diagnoses like cerebral insult due to vascular lesion [2] and compression of the trachea [3], the subclavian vessels [4], and the brachial plexus [5]; also, fatalities are documented due to a tracheoesophageal fistula [6].

An accurate diagnosis and appropriate treatment are therefore important for a good outcome [7]. Only few case reports or small case series exist in the current literature. These injuries are described as dislocation of the SCJ or epiphysiolysis of the medial end of the clavicle. The mechanism of injury is either a direct force applied to the medial clavicle or an indirect force due to an impact on the posterolateral aspect of the shoulder occurring often during contact sport activities [8].

The SCJ is functionally a saddle joint with a small surface providing only a poor congruence and containment. Therefore, a thick capsule and a strong ligamentous system guarantee joint stability. Spencer et al. showed on a cadaver model that the posterior capsule is the most important restraint for anterior and posterior translation in the SCJ [9]. Anterior dislocations occur nearly three times more often than posterior dislocations [10].

The understanding of the process of clavicular ossification is essential for an appropriate management of injuries around the SCJ in skeletally immature patients. The center of ossification of the medial end of the clavicle appears at the age of 18 to 20 years, and its growth plate fuses at the age of 20 to 25 years [11, 12].

**Figure 1:**
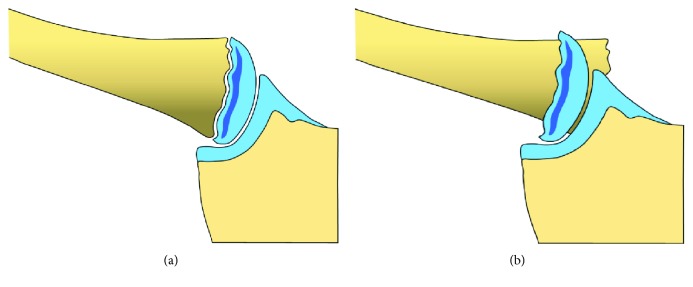
Sternoclavicular joint of an osseous immature person (a). Typical injury pattern: epiphysiolysis type Salter I with posterior displacement of the clavicular metaphysis (b).

Therefore, the physis of the medial clavicle is the weakest link in the SCJ and it is more likely to sustain a physeal separation than a true SCJ dislocation (Figures 1(a) and 1(b)). The strong ligamentous structures of the SCJ retain the cartilaginous epiphysis in place, and the metaphysis of the clavicle displaces posteriorly towards the retrosternal space. On standard radiographs, the diagnosis of a SCJ lesion can be missed easily. Therefore, the clinical assessment of local pain and swelling and SCJ instability are important. To confirm the diagnosis and evaluate additional retrosternal injuries, further analysis using a CT scan is mandatory. However, even with a CT scan, discrimination between true SCJ dislocation and displaced epiphysiolysis type Salter I or II can be difficult [13–17]. Often, the final diagnosis is made during surgery [18].

In the current orthopedic literature, there are just a few case reports or small case series reported of the rare entity of epiphysiolysis of the medial clavicle.

Our objective is to propose an appropriate management of epiphysiolysis of the medial end of the clavicle in the skeletally immature patient.

## 2. Cases

We report on three patients suffering from traumatic epiphysiolysis of the medial clavicle and discuss the surgical management and fixation techniques.

**Figure 2:**
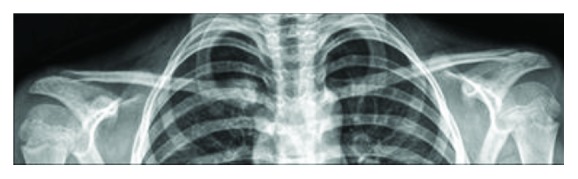
Preoperative anteroposterior radiographs revealing an asymmetric position of the medial clavicle on the right side.

**Figure 3:**
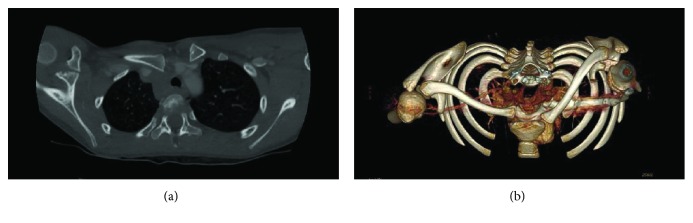
Axial (a) and three-dimensional reconstructed (b) computed tomography scans confirm the posteriorly displaced clavicle on the right side. Due to the absence of the corresponding centers of ossification of the medial clavicular epiphysis, discrimination from a pure posterior dislocation of the sternoclavicular joint is difficult.

In the first two cases—a 13-year-old boy and an 8-year-old girl, the mechanism of injury consisted in a lateral impact onto the involved shoulder while falling during a soccer play. In the third case of a 16-year-old boy, the mechanism was a direct blow in the anteroposterior direction on the upper thorax while practicing Judo. All three patients suffered from an instant pain in the right SCJ area. Clinically, no neurovascular lesions or dyspnea were present. Standard radiographs revealed an asymmetric position of the medial clavicle with posterior displacement of the medial end on the injured side (Figure 2). CT scan confirmed the diagnosis of epiphysiolysis of the medial clavicular epiphysis (Figures 3(a) and 3(b)). But, due to the absence of the corresponding centers of ossification of the medial clavicular epiphysis, discrimination from a pure posterior dislocation of the SCJ was difficult preoperatively, and final diagnosis was only possible during open surgery.

In the first child, an unsuccessful closed reduction was attempted prior to operation. The operation was performed under general anesthesia with the patient in supine position. A cushion was placed between the scapulae to exert tension on the anterior upper thorax. A 4 cm long skin incision was made in line with the clavicle from its medial end to the sternum. The periosteum, which was found intact anteriorly, was incised over the medial clavicle. The thick periosteal sleeve was disrupted posteriorly allowing posterior displacement of the medial clavicular metaphysis which was locked posterior to the manubrium. The medial clavicular epiphysis remained in place; thus, the lesion was classified as an epiphysiolysis type Salter I.

Reduction of the clavicle back in its periosteal sleeve was performed by gentle traction and the use of a pointed reduction clamp. Stabilization of the medial clavicular metaphysis was performed with a transosseous suture using a FiberTape® in a figure-of-eight fashion from the clavicular metaphysis to the anterior cortex of the sternum.

Due to the experience of the first case in which the clavicular metaphysis was trapped behind the periosteal sleeve, surgical technique was adapted for the second and third children.

**Figure 4:**
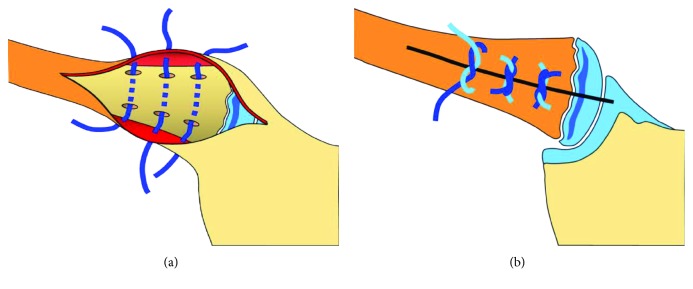
Surgical technique: fixation of the reduced clavicular metaphysis with three transosseous sutures (FiberWire®) on the anterior clavicular periosteum (a). The clavicular metaphysis is fixed, and the periosteum is closed by tightening the knots (b).

The skin incision was shorter, about 2 cm centered on the medial end of the clavicle. The platysma was just fenestrated. In both cases, the periosteal sleeve of the medial clavicle was found to be completely intact in its anterior part. It was incised longitudinally leaving intact the capsule of the SCJ. As seen in the first patient, the medial clavicular epiphysis stayed properly in place and the clavicular metaphysis was displaced retrosternally disrupting the posterior periosteum longitudinally. Open reduction was performed. Three drill holes were made in the superior and inferior cortex of the medial clavicular metaphysis avoiding perforation of the posterior cortex. Three FiberWire® were inserted in the holes, and the metaphysis was anchored directly to the strong and solid anterior clavicular periosteum (Figures 4(a) and 4(b)). Closure was completed in layers by additional adaptation of the anterior periosteum and the platysma.

Intraoperatively, in all three children, a good stability was achieved with the proposed suture techniques. No complications occurred during the operation. Facing the rare risk of severe vascular or pulmonal problems especially during the reduction maneuver, we assured backup by a thoracic surgeon in all cases.

Postoperatively, the patients were wearing a posterior figure-of-eight bandage for six weeks and free mobilization of the shoulder was allowed below the horizontal plane. At twelve weeks, all three patients were asymptomatic. The range of shoulder motion was symmetric; the body-cross test negative, and clinically the medial clavicle was stable. Radiographs revealed healing of the epiphysiolysis in correct length of the clavicle and correct position of the medial clavicular metaphysis. Also, at one-year follow-up, the patients remained asymptomatic and without any functional impairment, and all had returned to former sport activities.

## 3. Discussion

Actually, there is no consensus in the literature concerning the optimal treatment of this physeal fracture. Conservative treatment without anatomical reduction by thinking that the remodeling potential of immature bone would be able to restore correct anatomical position of the medial end of the clavicle is described in the past, but with reported complications like thoracic outlet syndrome by callus formation [19] and pneumothorax [20].

Denham and Dingley published probably one of the first reports of epiphyseal separation of the medial end of the clavicle. He favored an anatomical reduction to improve healing and decrease the number of poor results [21]. Closed reduction was recommended by several authors if there are no additional retrosternal injuries [16, 21–24]. However, the reported success rate was very diverging (Table 1). Often, an open reduction and internal fixation were required after a failed attempt of closed reduction or in case of residual instability [14, 23, 25–27]. Waters et al. [23] reviewed 13 medial clavicle injuries in skeletally immature patients and found 11 physeal fractures. They reported on instability after closed reduction in all cases and recommend immediate open reduction and fixation [23]. Laffosse et al. [14] reported a series of 13 patients with systematic failure of the attempted closed reduction. They also recommend open reduction with a stabilization procedure [14].

The methods described for stabilizing these physeal fractures are quite different including fixation by Kirschner wires [21], anterior plating [17, 28], or various suture techniques such as costoclavicular cerclage or tenodesis [14], repair of the costoclavicular and sternoclavicular ligaments [13, 23], transosseous fixation in a figure-of-eight manner of the clavicular metaphysis to the intact epiphysis [25, 29], or suturing the clavicle to the manubrium [18, 26, 30, 31]. The use of transarticular Kirschner wires has been abandoned due to the risk of intrathoracic wire migration [32].

Tennent et al. described a new stabilization technique [27]. After open reduction, they sutured the medial clavicle to the anterior periosteum and platysma as a single layer using absorbable sutures, which were passed through drill holes in the medial clavicle. At an average of nine months, their patients presented good functional results. Only one patient had to be reoperated six months after the initial procedure to remove a prominent suture knot and to revise the scar.

We are convinced that it is important to distinguish between a real dislocation of the SCJ and a physeal fracture of the medial end of the clavicle. Osseous immature patients suffer rather from physeal fracture type Salter I or II then from a pure dislocation of the SCJ. Nevertheless, these two entities are not every time properly discriminated in the literature, and in case of successful closed reduction and nonoperative treatment, the exact diagnosis will remain uncertain. We should keep in mind that in epiphysiolysis of the medial clavicle, the strong periosteal sleeve plays an important role. Corresponding to lateral physeal fracture, the clavicle peels off from its physis and periosteal sleeve, which is just disrupted longitudinally [33].

In all our three cases, the medial epiphysis remained anatomically in place, and the clavicular metaphysis was displaced posteriorly to it into the retrosternal space. In all three cases, the periosteal sleeve was only disrupted posteriorly allowing the posterior penetration of the medial clavicular end.

Due to its small dimension, the medial clavicular epiphysis itself does not offer sufficient grip for anchoring sutures. Thus, two different options to restore stability of the medial clavicle are available. The first one consists in a transosseous figure-of-eight suture of the clavicular metaphysis towards the anterior cortex of the sternum. The second one is comparable to the technique described by Tennent et al. [27]. The anteriorly intact periosteal sleeve is incised, the clavicular metaphysis reduced back into its sleeve, and held in place by means of three transosseous sutures from the clavicular metaphysis on the anterior clavicular periosteum. In contrast to the technique described by Tennent et al., we did not include the platysma in the suture allowing a better coverage of the knots by the platysma. In addition, instead of PDS, we used FiberWire®, thus creating less prominent knots.

We attempted a closed reduction in the first patient which failed because the metaphysis was stucked outside the periosteal sleeve posterior to the epiphysis as seen later intraoperatively. This finding agrees with the literature review shown in Table 1, which suggests that a closed reduction is rarely successful. Even if the patients do not present further initial symptoms, in our opinion, an immediate (up to 6 h) open reduction and fixation are indicated to avoid injuries of retrosternal structures by either the instable fragment or callus formation.

In contrast, when physeal fracture of the medial clavicle with anterior displacement occurs, a conservative treatment is often recommended. In this case, the periosteal sleeve is just disrupted anteriorly and stays intact posteriorly, which protects the mediastinal structures.

The advantage of the described methods is that neither the fragile epiphysis nor the SCJ is violated, the retrosternal structures are safe, and there is no hardware irritation. Mechanically, the clavicular metaphysis buttresses against the clavicular epiphysis, and the transosseous sutures into the anterior periosteum prevent the posterior redislocation. The bone is also loaded in compression, and the restored anterior periosteum plays the role of a tension band.

We practiced both stabilization techniques. Both had excellent clinical and radiographical results at one year. Nevertheless, we recommend the second one stabilizing the clavicula on the anterior periosteum, because it is technically simpler and safer referring to possible iatrogenic retrosternal injuries.

## Figures and Tables

**Table 1:** Literature review (since 2000).

Author, year	*n*	Age	Closed reduction	Definitive treatment
Goldfarb et al., 2001 [25]	6	7–16	6 failed	6 ORIF osteosuture
Waters et al., 2003 [23]	11	13–17	3 failed	11 ORIF osteosuture
Gobet et al., 2004 [16]	3	8–15	1 failed	1 ORIF osteosuture
			2 successful	2 nonoperative
Hofwegen and Wolf, 2008 [26]	2	17–20	1 failed	2 ORIF osteosuture
Laffosse et al., 2010 [14]	13	15–20	5 failed	13 ORIF different techniques
Tennent et al., 2012 [27]	7	14–19	7 failed	7 ORIF osteosuture
Garg et al., 2012 [13]	1	12	1 failed	1 ORIF osteosuture over SCJ
Gil-Albarova et al., 2012 [34]	1	11	1 successful	1 nonoperative
Koch and Wells, 2012 [29]	1	14	0	1 ORIF osteosuture
Lee et al., 2014 [35]	20	13–19	2 failed	18 ORIF osteosuture
			2 successful	2 nonoperative
Ozer et al., 2014 [36]	1	16	1 successful	1 nonoperative
Tepolt et al., 2014 [18]	6	7–17	2 failed	6 osteosuture over SCJ
Perdreau et al., 2014 [37]	1	16	1 failed	1 osteosuture over SCJ
Krantzow, 2015 [38]	1	17	0	1 osteosuture
Kassé et al., 2016 [39]	3	16–19	0	3 osteosuture or cerclage SCJ
